# Determinants of malnutrition among urban slum children in Bangladesh

**DOI:** 10.1186/s13561-015-0059-1

**Published:** 2015-07-16

**Authors:** Adnan M S Fakir, M Wasiqur Rahman Khan

**Affiliations:** Department of Economics and Social Science (ESS), BRAC University, Dhaka, Bangladesh

## Abstract

**Background:**

This paper analyzes the role of child, maternal and household variables on weight-for-age nutritional status of children in the largest urban slum of Bangladesh.

**Methods:**

We use anthropometric weight-for-age nutrition status of children for an ordered logistic analysis. Our dataset gives us the advantage of segregating health knowledge into three indices: health-seeking practices index, child health precautions index and medical cost knowledge index, which are used as covariates to understand the role of health knowledge towards child health. Gender specific regressions are also run to understand male and female children nutritional function differences.

**Results:**

Per capita income significantly improves child health but household assets do not, casting doubt on the robustness of permanent income. After controlling for health knowledge and health-seeking behavior, the remaining impact of maternal education on child health is no longer significant. Health knowledge indices significantly improve child health albeit differentially. While male children are more sensitive to "child health precautions" and "medical cost knowledge", female children are more sensitive to "health-seeking practices".

**Conclusion:**

Role of health knowledge on child health carries a significant portion of the education effect. Policy makers looking to improve the nutritional status of female children vis-a-vis male children in study area, should promote programs focusing on health-seeking practices.

## Background

The economic improvements in South Asian countries over the past decade have not been adequately reflected in improvements in child nutrition [17, 26, 52]. Malnutrition not only makes the child more vulnerable to morbidity and mortality [22, 36], but has been linked to poorer educational attainment [30], delayed mental development [37] and lower intellectual and physical abilities in adult life [50].

The extent of malnutrition is shown to be much more severe in slums compared to children living in developed city areas, and sometimes even compared to rural regions [5, 25, 43, 54]. Primarily using an ordered logistic model, this paper analyzes nutritional status for children in the largest urban slum of Bangladesh. This paper focuses on the role of individual, maternal and public health practices by the household on malnutrition.

There are conflicts regarding the role of each of these attributes on child health. Where household income was overemphasized in the past, the significance of other household attributes, such as female literacy [10], autonomy [16], and maternal health knowledge [27] have been duly recognized.

The majority of existing studies either use nutrient calorie intake or anthropometric measures for malnutrition analysis. Children are generally identified as malnourished within the first two years of their life, even though the process is a cumulative effect that spans generations [56]. Olack (2011) [41] and Alam (2011) [41] report 37 to 47 months and 48 to 60 months, respectively, as being the age ranges with the highest prevalence of under nourishment. Poor fetal growth and/or undernourishment in the early child years lead to irreversible damage causing shorter adult height as well as lower weight [55]. Height-for-age and weight-for-age thus measure child growth relative to its potential, reflecting chronic and acute nutritional deprivation [34]. Our dataset contains nutritional status based only on weight-for-age information; hence we keep our study limited to acute wasting measures.

Fenske (2013) [23] and Goudet (2015) [29] both extensively identify the causes of malnutrition in children, keeping in conjunction to the UNICEF malnutrition model, segregating them as immediate (individual), intermediate (individual and household) and underlying (maternal, household and regional) factors. Nutrient deficiencies *in utero* [22], inadequate nutrition after breastfeeding and early life infections are deemed as immediate causes. Driving these immediate causes are the intermediate and underlying factors which include, but are not limited to child care practices, food security, household wealth, maternal education, health services, hygiene practices and sanitation conditions. All of these are of course again embedded in the larger socio-economic, environment and political sphere. Goudet, Faiz, Bogin and Griffiths (2011) [28], in particular, performed focus group discussions (FGD) in urban slums in Dhaka, Bangladesh and determined flooding to be an important determinate of child health.

Malnutrition continues to be a major problem for Bangladesh. According to the 2011 Bangladesh Demographic and Health Survey (BDHS) [8], 41 % of children under five were identified to be stunted, a marginal decrease compared to 43 % identified in BDHS 2007 [7]. Similarly underweight estimates for children under five linger at 33 % for 2011 compared to 41 % identified in 2007. Multiple studies have previously looked into various determinants of child health in Bangladesh, albeit few in specifically urban slum settings. Greater household wealth, urban locality, greater birth interval, earlier birth order, timely breastfeeding initiation, higher maternal age, increased number of antenatal visits, greater exposure to mass media, lower diarrheal incidence, as well as mothers’ being employed, have been linked to lower malnutrition among children under five in Bangladesh [1, 2, 6, 19, 39, 45–47, 49].

While the majority of the aforementioned studies have looked into malnutrition at a national level, there is a paucity of literature focusing on urban slums. This study contributes to the literature by focusing on the intermediate and underlying factors of household wealth, maternal education, child care practices and hygiene behavior as determinants of child health specifically in an urban slum setting in Bangladesh.

The Korail Nutrition Pilot Study (KNPS) dataset used for this study was collected by BRAC University in 2011 from Korail slum settlement, Dhaka, using a systematic random sampling method following Alam (2009) [3] for a pilot study on nutrition. Korail is the largest slum settlement in Bangladesh and captures a good deal of the diversity present in Bangladeshi urban slums. This paper makes use of the nutrition survey data collected from 174 children below the age of five. Relevant household individuals were enumerated for selected individual, child and household level data. A key aspect of the data is three sets of questions regarding practiced household public health behavior and awareness. We make use of these to construct indices to determine the impact of maternal knowledge through the public health practices pathway on child health.

The structure of the remaining paper is set up as follows. Section two describes the study location characteristics and the descriptive statistics of the data. The section continues to introduce the primary covariates of the model, presents arguments for their plausibility as determinants where necessary and provides a succinct discussion of previously documented impacts of the covariates. Section three builds the econometric model with a discussion of the findings and section four concludes the paper with a summary and recommendations.

## Methods

### Location characteristics

The definition of a slum is considered to be same as set forth by Islam, Mahbub, Nazem, Angeles & Lance (2005) [31], “A slum (is) thus defined as a neighborhood or residential area with a minimum of 10 households or a mess unit with at least 25 members with four of the following five conditions prevailing within it: predominantly poor housing; very high population density and room crowding; very poor environmental services, particularly water and sanitation facilities; very low socioeconomic status for the majority of residents and lack of security of tenure.”

The Dhaka Metropolitan Area of Dhaka city, the capital of Bangladesh, has been estimated to have a population of 9.1 million of which 3.4 million or a large 37.4 percent are slum dwellers. For this study, the largest informal single slum in the Dhaka (and also in the country), the Korail slum settlement, was selected. The Korail slum settlement covers an area of approximately 85 acres of encroached land with an estimated population of 80,000 [13].

Located near the high-end residential and commercial areas of Dhaka it attracts low income people engaged mostly in service jobs such as cleaners, household helpers, rickshaw pullers and ready-made garments workers [32].

High population density without established services exemplifies the settlement. The majority of households are tin-sheds often sharing a single cooking place and shared cemented pit or hanging latrine, often unhygienic. With the local Gulshan-Banani Lake on the southern and eastern boundary, the slum is especially prone to water-borne diseases. Water logging following severe rainfall is also common.

Lack of security of tenure and consequently threat of eviction is one of the major concerns. This has caused reluctance among the authorities to provide regular public service facilities. Jabeen, Allen and Johnson (2009) [32] note that “inhabitants living as long as even 20 years are unwilling to invest in improving the living condition,” due to the threat of eviction. According to Biplob, Sarkar and Sarkar (2011) [13] around 57 % respondents use the national water supply from the municipal authority for their drinking water and other purposes while 43 % respondents use shallow tube-wells. There is electricity supply but cooking is done on bought firewood.

Several organizations have been working in the Korail slum to improve their public health behaviors through maternal knowledge and awareness in the past years. Also, it is to be noted, householders in the slum often walk notable distances to collect tapped water for drinking instead of using open and stagnant tanks which can be considered a behavioral improvement in the area, albeit it remain to be tested empirically. While indices have been prepared to reflect selected behavioral attributes acting as covariates, this specific hypothesis remains untested.

### Socio-economic characteristics

Table 1 provides a quick overview of selected descriptive statistics of the KNPS dataset collected from the Korail slum settlement in 2011. A more detailed discussion of the variables and their role in child health is taken up later in in this section when we introduce our variables.

**Table 1:** Selected socioeconomic characteristics

	Nutrition Status			All	Pearson Chi-square *P*-value
	Normal	Moderately Malnourished	Severely Malnourished		
**Child < 5 Characteristics**					
Frequency	28.2	20.7	51.1	-	-
Female	36.7	50.0	58.4	50.6	0.051^a^
Mean Age	33.9	36.5	27.9	31.3	0.055^a^
Illness Incidence	34.7	55.6	40.4	42.0	0.144
**Maternal Characteristics**					
Literacy	0.857	0.500	0.573	0.638	0.062^a^
Working	1.184	1.139	1.056	1.109	0.316
**Household Characteristics**					
Family Size	4.55	4.58	4.56	4.56	0.896
Income	7,338	5,757	5,144	5,889	0.040^a^
Poverty	44.9	50.0	67.4	57.5	0.022^a^
Asset Index	0.099	0.073	0.084	0.086	0.544
Owns Land	46.9	36.1	33.7	37.9	0.299
Has Saving Habit	26.5	22.2	20.2	22.4	0.696

*Maternal Education* is coded 0 for no education, 1 for some education but less than primary, 2 for higher than primary but less than secondary, 3 for secondary and greater; *Poverty* defined as households earning less than USD 2 (BDT 167) per day; *Household (weighted) Asset Index* created from ownership of common household assets as follows (weights in brackets set according to approximate local prices): radio (1), mobile (2), cycle (5), boat (10), rickshaw (25), bedding (1), almira (1), chair/table (0.5), fishing net (1), sewing machine (2), cow/ox (10), goat (3), hen/duck (0.5) and TV (5). For example, if a household owned only a rickshaw, bedding and cow, the weighted asset index value for that household would be 36/67 = 0.537

^a^ = significant at the 10 percent level

Child nutrition status was noted into three ordinal categories that measures child growth relative to potential. The standardized weight curves, from birth to five years old, provided by the World Health Organization (WHO) was used. Convention on malnutrition categorization was followed with children >2.0 standard deviation but <3.0 standard deviation below the median reference categorized as moderately malnourished, and children >3.0 standard deviation below the median reference categorized as severely malnourished.^1^

In our sample, out of 174 observations only 28.2 percent were well-nourished. About 20.7 percent were moderately undernourished while a majority 51.1 percent is severely undernourished. Expectedly, incidence of malnourishment is much higher in the slums. We see a higher percent of female children under severe malnourishment (58.4 percent) as opposed to normal (36.7 percent), indicating potential sex bias in nutrient uptakes. Comparatively younger cohorts of children also belong to the severely malnourished category. We also note a weak correlation between family size and child heath as noted by earlier studies [35].

Maternal literacy and number of working mothers both decrease with malnutrition, while the latter has a high Pearson chi-square p-value. In a study in Benin, Reed, Habicht and Niameogo (1996) [48] find a negative association of working mothers with malnutrition as working mothers spends fewer hours in childcare at home. However the opposite can also be true where working mothers, by earning higher income, provide more household food, resources and better quality of care [9]. Although not significant, it seems our data leans towards the latter.

If we consider saving habit as an inclination to take security measures against shocks, severely malnourished households have the least habit. Saving habit is however not statistically significant with levels of nourishment. Mean income falls with the levels of malnutrition, as proportion of households in poverty increases, with 67.4 percent of severely malnourished child households living under two dollars a day.

The increasing poverty with malnutrition is also reflected through land holdings. Proportion of households owning at least some land in their villages is lowest for the severely malnourished category. It is noteworthy that child illness incidence is not the highest among the severely malnourished (although it is lowest for the well nourished), reflecting the causality problem of illness with malnutrition; while it is not necessarily a cause of malnutrition, being malnourished can cause illness.

Before we analyze the empirical evidence we consider the different arguments on the role of variables related to child health. Malnutrition is an intricate socioeconomic phenomenon with much conflicting empirical outcomes that demands cautious interpretation. In the following we introduce our explanatory variables as well as their underlying analytical arguments from the literature.

### Household income

As expected, income plays an important role in child health determination. While the causal effect of income on child health has been questioned by several authors [26, 33], unarguably a large portion (if not all) of income's role on child health operates through various other factors. Many inputs, such as food intake, household sanitation, quality of medical care received that are correlated with child health depend on income, which the variable reflects to an extent. Income on the other hand also depends on level of education. In other words it also reflects an income effect portion of the total education effect [53]. Ideally one should control for these (unobserved) heterogenic factors to better understand determinants of child health, but limitations on data often do not permit so.

The instrument of choice used to measure income is crucial in analysis. Although permanent income or household expenditure tends to be better estimates of resource availability they are difficult and expensive to measure. Hence even though current income tends to have transitory components it is usually used as a proxy. Thomas, Strauss and Henriques (1991) [53] point out some measurement problems related to current income, namely (i) respondents may be unwilling to disclose their income, (ii) income from self-employment is hard to measure and (iii) if only the mother of the household is surveyed, she may not know the total income of the household. It is likely that the measure of current income in our dataset also faces these common problems.

Income is endogenous in nature which gives rise to the possibility of simultaneity bias. For example parents with children who are ill may decide to work more hours to pay for better medicine, thus earning more income. Because such negative shocks would increase household income in such cases, the impact of income on child health would be underestimated [26]. To avoid such simultaneity bias one can use non-labor income or value of household assets in place of current income.

Since our dataset does not allow us to estimate permanent income, we use two instruments to proxy income. The first is current income per capita while the second is a household assets index prepared from approximated values of household assets. The latter can be considered a crude longer-term income measure which also tackles the simultaneity bias to an extent.

### Maternal education & health knowledge

In societies where mothers are the main caregivers of the child, maternal education has been shown to have a stronger and significant effect on child health than paternal education [11, 14, 40]. Ever since the causal pathways between mother's education and child health has been in question.

Past studies have found a strong correlation between maternal education and indicators of care, such as better sanitation practices, better child feeding, timely immunization etc. [14, 15, 21]. Unger (2013) [54] and Cebu Study Team (1991) [51] find that educated mothers are better at making choices in caring practices and recognizing threats to the health of their children. The causal link is that mother's education induces behavioral changes which lead to lowered prevalence of childhood diarrhea.

Glewwe (1999) [27] proposed that maternal education operate on child health primarily through three mechanisms: (i) formally taught health knowledge, (ii) numeracy and literacy skills that brings about cognitive development, and (iii) by making women more receptive to modern medicine. Using a Moroccan dataset, he concluded that health knowledge, acting as a mediator, does improve child health while numeracy and literacy skills promote uptake of health knowledge outside the classroom bringing about behavioral change. The finding is consistent with that of Desai and Alva (1998) [21] and Frost, Froste and Haas (2005) [24] who also concluded that maternal education influences health seeking behavior which in turn influences child health.

In a study in Bangladesh, Bhuiya, Streatfield and Meyer (1990) [12] identify specific types of health knowledge associated with higher levels of education, including (i) washing hands after latrine use, (ii) use of oral rehydration therapy to treat diarrhea, (iii) awareness of the benefits of boiling water, and (iv) contagions as a means of spread of disease. Such health knowledge can lead to the behavioral changes that result in better household health-seeking practices and subsequently better child health.

In addition to levels of maternal education, the dataset at our disposal contains similar behavioral information that permits us to construct binary 'deprivation' indices of maternal health knowledge. We break down health knowledge into three components: (i) health-seeking practices, (ii) child health precautions, and (iii) medical cost knowledge. Table 2 describes deprivation conditions for each of the indices as well as how each index is prepared from specific questionnaire inquiries. Note that the binary indicators are summed for each index and the deprivation cutoff value is then applied. For example if a household does not practice more than 2 out of the 8 household health-seeking practices, the household is considered deprived in that component.

**Table 2:** Description of indices

Description of index indicators	Deprivation condition	Sample proportion deprived
**Health-seeking practices**		
- Whether drinking water is boiled before consumption	Any child household not practicing more than two of the indicators	44.8
- Whether water from tap or tube well is used to wash dishes		
- Whether water from tap or tube well is used to take showers		
- Whether hands are regularly washed before eating		
- Whether hands are regularly washed after use of latrine		
- Whether nails are cut and cleaned at least once a month		
- Whether teeth are brushed at least once a day		
- Whether there is any stagnant water around the household		
**Child Health Precautions**		
- Children aged 9–59 months who have not received Vitamin A supplementation in the previous 6 months	Any child deprived in more than one of the indicators	37.9
- Children aged 12–59 months who have not received at least one dose of measles immunization		
- Children living in households with non-iodized salt		
- Children aged 6–59 months with no adult support for more than 5 hours in a day		
**Medical Cost Knowledge**		
Local treatment estimate for the following diseases (± BDT 500)	Any child household not aware of more than two of the treatment costs	64.4
- Diarrhea		
- Jaundice		
- Typhoid		
- Dengue		
- Cholera		

The health seeking practices index provides an indicator of the hygiene practices of the household. Similarly, the child health precautions index acts as a proxy for essential child care practices. Finally, the medical cost knowledge index, while perhaps not an intermediate or underlying determinant of child health, is meant to capture awareness and knowledge about planning for child health. Mothers who are keenly interested to remain aware and well prepared about future potential child health problems will keep themselves updated regarding the treatment costs. This index is meant to reflect such an innate behavioral trait and is ideal to be controlled for.

Some limitations should also be pointed out. Innate characteristics of mothers, which are often hard to measure, also influence the role of education on child health. Thomas, Strauss and Henriques (1991) [53] showed that almost all the impact of maternal education on child survival could be explained by how easily the mother's could access information, such as read newspapers, watch TV and/or listen to radio in Brazil, indicating the importance of 'general awareness' of the mother along with health knowledge. Although the medical cost knowledge index partly captures specific health awareness, none of our indices capture media exposure and remains uncontrolled for which can lead to an overestimation problem.

Part of the effect of health knowledge also suffers from endogeneity problems. Knowledge about health and nutrition can be passed down over generations, from grandmothers to mothers, as a form of 'genetic endowment'. Similarly, a mother whose child has diarrhea is more likely to know about oral rehydration salts (ORS) than a mother whose child does not [38]. Thus endogeneity occurs when such unobserved 'genetic endowments' to be sick or short are causally related to both knowledge and child nutrition.

Glewwe (1999) [27] uses the heights of parents as an instrument to tackle this problem by adjusting for child's unobserved health endowment, since taller parents are likely to have better health endowments. Wolfe and Behrman (1987) [11] used data mother's siblings to control for unobserved family fixed effects. We do not have data for parents' heights, or any appropriate instrument to control for genetic endowments and ignoring this can lead to an underestimation of the impact of knowledge.

We also note that our medical cost knowledge index likely suffers from some extent of endogeneity. There are two primary ways through which a mother living in an urban slum would be able to know about specific medical treatment costs: First, mothers who have gone through the ordeal of the disease in question with their children would know its treatment costs, and second, simply through talking to neighbors out of interest or to NGO workers in the area advocating financial planning for child health. What is of concern is the former reasoning. Mothers of children with poor health status are more likely to know more about medical costing than mothers with children of good health standing. Conversely, mothers with more medical cost knowledge are likely to take better care of their children via better financial planning and heightened awareness. Thus a causality problem remains.

Finally it is worth mentioning that there are several other pathways through which maternal literacy can influence child health; our three indices only capture a portion of the total effect. Alderman, Hoogeveen and Rossi (2005) [4] report, that in addition to individual level variables, maternal education may also operate through community factors. Das Gupta (1990) [18] concludes that women with greater autonomy and household decision-making power have healthier children. These are components that our model does not consider so we do not regard our model to be inclusive. However our expectation is to better understand the roles of the three indices to child health in Bangladesh's urban slums.

### Siblings

The number of children a family is willing to have is an endogenous decision process [26]. Not only does the decision to have a second child depend on the health of the firstborn but educated parents normally prefer to have fewer children [44]. However David, Moncada and Ordonez (2004) [20] point out that if family planning is not adequately practiced, this endogeneity is likely to be small. Considering such is still the case in the Korail slum settlement, we include a dummy for having more than one sibling in our model. A likelihood ratio test tells us that adding this variable provides a statistically significant improvement in model fit (p-value of 0.0052).

### Interaction terms

As discussed previously, there is an extent of correlation between some of our variables, which could lead to biased estimates. In order to control for this possible bias we include a set of interaction terms. As literate mothers are more likely to belong to wealthier households, we include an interaction term between female education and per capita income and between female education and household assets index. Similarly, as previous studies have shown, educated mothers are more likely to perform health seeking practices and take child health precautions [51, 54]. To control for this we also include interaction terms of both health seeking practices and child health precautions with female education.

## Results

### Ordered logistic estimates

We use an ordered logistic model in accordance to the ordinal nature of the data on child nutritional status. The dependent variable considers three levels of nourishment: 0 for normal growth, 1 for moderately malnourished and 2 for severely malnourished.

Table 3 reports coefficients for five model variants sequenced from models (1) to (5). Each of the five model variants reports the odds ratio (OR) from five ordered logistic regressions, with the levels of nourishment being the dependent variable. The base model (1) provides the effect of covariates with socio-economic factors without controlling for any of the indices. The subsequent models (2) and (3) build on the base model by adding the child health precaution index, the health seeking precautions index and the medical cost knowledge index, along with their relevant interaction terms as discussed under section 2.6. The difference between models (2) and (3) is the choice of proxy reflecting household wealth; model (2) uses the household asset index whereas model (3) uses per capita income. Finally models (4) and (5) disaggregate the model by gender; model (4) is for male children only and model (5) is for female children only.

**Table 3:** Odds ratio (OR) estimates of child nutritional status (standard errors)

Variable	Model (1): Base	Model (2): Asset Index	Model (3): Per Capita Income	Single score test (*p*-value)	Model (4): Male Only	Model (5): Female Only
Child Sex (1 = Female)	0.729^a^ (0.307)	0.748^a^ (0.339)	0.640^a^ (0.348)	0.1239	-	-
Child Age (in months)	−0.021^a^ (0.009)	−0.023^a^ (0.009)	−0.026^b^ (0.009)	0.0721	−0.048^b^ (0.015)	−0.002 (0.014)
Per Capita Income	−0.471^b^ (0.176)	-	−0.519^a^ (0.278)	0.7717	−0.699^a^ (0.390)	−1.201^a^ (0.542)
Household Asset Index	-	−1.643 (2.540)	-	-	-	-
Maternal Education	−0.425^a^ (0.254)	−0.937^a^ (0.488)	−0.033 (0.565)	0.2254	−1.648^a^ (0.908)	1.317 (1.094)
Have Older Siblings (1 = Yes)	−0.555^a^ (0.330)	−0.785^a^ (0.359)	−1.046^b^ (0.384)	0.4068	−1.551^a^ (0.610)	−1.403^a^ (0.604)
Child Health Precautions Index	-	1.893^b^ (0.553)	1.786^b^ (0.554)	0.1445	1.883^a^ (0.956)	2.179^a^ (0.885)
Health Seeking Practices Index	-	0.649 (0.469)	0.837^a^ (0.475)	0.9571	0.252 (0.751)	3.123^b^ (0.823)
Medical Cost Knowledge Index	-	1.081^b^ (0.364)	1.293^b^ (0.375)	0.6970	1.510^a^ (0.646)	1.221^a^ (0.583)
Maternal Education × Per Capita Income	-	-	−0.242 (0.245)	0.2456	−0.237 (0.410)	0.893 (0.653)
Maternal Education × Household Asset Index	-	1.316 (2.094)	-	-	-	-
Maternal Education × Child Health Precautions Index	-	0.161 (0.568)	0.640 (0.639)	0.2784	1.193 (1.172)	−0.209 (0.995)
Maternal Education × Health Seeking Practices Index	-	0.077 (0.533)	−0.268 (0.573)	0.2233	−0.254 (1.075)	−2.453^a^ (0.975)
L_0_	−178.4811	−178.4811	−178.4811	-	−90.9896	−84.4876
L	−165.7001	−147.2901	−139.4184	-	−58.6590	−62.3075
LR	25.56^b^	62.38^b^	78.13^b^	-	64.66^b^	44.36^b^
Observations	174	174	174	-	86	88

*Per Capita Income* is scaled down by a factor of 1,000; *Household Asset Index* is prepared as described under table 01 caption; *Maternal Education* is coded 0 for no education, 1 for some education but less than primary, 2 for higher than primary but less than secondary, 3 for secondary and greater; *Child Health Precautions Index, Health Seeking Practices Index and Medical Cost Knowledge Index* are prepared as described in table 02 coded as 0 if not deprived and 1 if deprived

*LR = 2(L-L*_*0*_ *)* is the log likelihood ratio statistic with a chi-square distribution where *L*_*0*_ is the restricted log-likelihood function and *L* is the log-likelihood function

^a^ = significant at the 10 % level

^b^ = significant at the 1 % level

In order to check the proportional odds assumption of the model, the chi-square test was employed. The chi-square value of the overall model is 12.49 with a prob > chi2 = 0.3281 so the proportional odds assumption is not violated. Since the score test can sometimes be anticonservative, we also conduct single score tests for the explanatory variables. The results of the single score test for the per capita income model (3) are presented in the column next to it and indicate that individually also, none of the variables violate the proportional odds assumption at five percent significance level. Between the household assets model (2) and the per capita income model (3), the likelihood ratio suggests that the goodness of fit is best when we consider per capita income as a proxy for household wealth, that is model (3) is considered the better of the two models.

Note that the covariates can be theoretically differentiated into three categories: the variables of primary interest (proxies of income and maternal education), socio-economic control variables (child sex, child age and whether the child has older siblings) and our three indices of primary interest reflecting health knowledge and health seeking practices.

The coefficient for per capita income under model (3) is negative and significant (OR = −0.519; p-value = 0.061), that is children living in households with more per capita income are less likely to be malnourished. Although the coefficient for the asset index under model (2) is also negative, it is insignificant (OR = −1.64; p-value = 0.518). Since we do not have permanent income or expenditure data, we cannot affirm the role of permanent income on child health. Furthermore, as mentioned earlier, our constructed asset index is somewhat of a rudimentary nature. However, if we do accept it as crude proxy, permanent income is found to be an insignificant factor in determining child health, a result that has also been suggested by Pal (1999). However, given the nature of our asset data, this finding should be taken cautiously, and we will focus on the per capita income model (3) for the remaining of the analysis. It is to be noted however that the remaining explanatory variables maintain the same direction in both models (2) and (3) indicating to some degree the robustness of the result.

Child age is negative and significant for our sample meaning malnourishment is higher for younger children and falls with age. The result is consistent for the all three models (1), (2) and (3). We also find a similar significant relationship with the number of siblings^2^ (OR = 0.150; p-value = 0.083). This is in contrast to the finding from Glewwe (1999) [27] who find a negative relationship. Glewwe (1999) [27] provided the rationale that with fewer children mothers are able to allocate more time and health inputs per child. In fact such a relationship is what we initially expected.

However, based on the results we reason older siblings help in looking after the young either through increased care or by earning in our study location. It is a common practice in the slums for the elder children to help look after the young and even in household activities. To confirm this, we replaced the number of siblings variable with a binary to indicate whether a child has older siblings and find a statistically significant result, as reported in Table 3 (OR = −1.046; p-value = 0.006 for the per capita income model). Following the same procedure for gender specific regression models (4) and (5), this also resulted in a statistically significant relationship, for both the male (OR = −1.551; p-value = 0.011) and female (OR = −1.403; p-value = 0.020) children. Marginal effects at the mean tell us that having an older sibling means that the child is 22.8 percent less likely to be severely malnourished while 15.5 percent more likely to be normal and 7.4 percent to be moderately malnourished, all statistically significant.

One advantage of retaining the base model (1) in the results table is that it lets us compare how much of the effect of maternal education on child health is soaked by adding our indices. While the odds ratio of maternal education is large and significant (OR = −0.425; p-value = 0.094) for the base model (1), it is small and insignificant (OR = −0.033; p-value = 0.953) for the per capita income model (3) which keeps the indices and interaction terms as covariates. It can be argued that the indices and the interaction terms capture a portion of the 'total' education effect. Thus, after controlling for child health knowledge and health-seeking behavior, the remaining impact of maternal education on child health is no longer significant.

The child health precautions index (OR = 1.786; p-value = 0.001) and the medical cost knowledge index (OR = 1.29; p-value = 0.001) are both positive and highly significant in the per capita income model (3): children are more malnourished in households that are deprived in taking child health precautions and are less knowledgeable regarding medical treatment costs. This is also consistent in the household assets model (2). The health seeking practices index, in contrast, is only significant (OR = 0.837; p-value = 0.078) in the per capita income model (3).

Child sex is positive and significant for all the three models (1), (2) and (3). So, other things remaining the same, female children are more likely to be malnourished than male children. Pal (1999) [42] suggested that male and female children follow separate nutritional functions and in view of the last result, we run separate gender specific regressions. Given that per capita income model provided a better fit, and for reasons explained earlier, we use per capita income as our proxy for household wealth instead of the household asset index. The results are provided in models (4) and (5) capturing the differences in male and female nutritional functions. Child age, per capita income, maternal education, having older siblings, child health precautions index and medical cost knowledge index, all significantly affect male children. For female children, per capita income, having older siblings, child health precautions index, health seeking practices index and the medical cost knowledge index are significant. The gender unified models (1), (2) and (3) fail to capture this complexity between male and female children nutritional functions.

While older male children face less malnutrition (OR = −0.048; p-value = 0.002) we cannot say so conclusively for the female children due to child age being insignificant (OR = −0.002; p-value = 0.915). Nutritional status for both genders improves with increased per capita income and having older siblings. Even after controlling for child health precautions and health seeking practices, maternal education is significant for male children (OR = −1.648; p-value = 0.070). Although maternal education is insignificant for female children (OR = 1.317; p-value = 0.229), it is interesting that the variable has opposite effects for the two genders: holding everything else the same, educated mothers improve the nutritional status of male children but lowers that of female children. Pal (1999) [42] also finds similar results in rural India using the World Institute of Development Economics Research (WIDER) dataset.

Finally, while both the child health precautions index and the medical cost knowledge index are significant for both genders, the health seeking practices is highly significant (OR = 3.123; p-value = 0.000) only for female children. In other words, for our study location, female children living in households that are deprived in health-seeking practices are relatively more malnourished, while the same cannot be conclusively said for male children.

### Gender specific marginal effects of covariates

The ordered logistic regression allows us to compute the marginal effect of each covariate on child health separately for normal, moderately malnourished and severely malnourished children. In order to further understand the differential nutritional functions by gender, we do this for the male and female regression models (4) and (5) from Table 3. Table 4 thus reports the effect of the covariates on the probability of being normal, moderately malnourished and severely malnourished separately for male and female children.

**Table 4:** Marginal effects of explanatory variables on child health for male and female children

Variable	Normal		Moderately Malnourished		Severely malnourished	
	Female	Male	Female	Male	Female	Male
Child Age	0.000	0.010^b^	0.000	0.001	−0.000	−0.011^b^
Per Capita Income	0.122^a^	0.141^a^	0.161^a^	0.013	−0.283^a^	−0.154^a^
Maternal Education	−0.134	0.332^a^	−0.177	0.031	0.311	−0.363^a^
Have Older Siblings (1 = Yes)	0.143^a^	0.314^a^	0.175^a^	0.009	−0.318^a^	−0.323^b^
Child Health Precautions Index	−0.213^a^	−0.320^a^	−0.248^a^	−0.106	0.461^b^	0.426^a^
Health Seeking Practices Index	−0.362^b^	−0.050	−0.272^b^	−0.006	0.634^b^	0.056
Medical Cost Knowledge Index	−0.136^a^	−0.331^a^	−0.150^a^	0.044	0.286^a^	0.287^b^
Maternal Education × Per Capita Income	−0.091	0.048	−0.120	0.004	0.211	−0.052
Maternal Education × Child Health Precautions Index	0.021	−0.024	0.028	−0.022	−0.049	0.263
Maternal Education × Health Seeking Practices Index	0.249^a^	0.051	0.330^a^	0.004	−0.579*	−0.056

^a^ = significant at the 10 % level

^b^ = significant at the 1 % level

The marginal effects supplements the inferences made from the parameter estimates. An increase in one unit of per capita income (BDT 1,000) decreases the probability of being severely malnourished by 28.3 percent for female children and 15.4 percent for male children. Thus while per capita income helps all children to step out of malnutrition, it helps female children more. However an increase in one unit of education decreases the probability of being severely malnourished by 36.3 percent for male children while increases the probability by 31.1 percent for female children (the latter is insignificant).^3^ This once again reflects the dipartite effect of maternal education on child health in our study location.

While interpreting the estimates on the child health precautions, health seeking practices and medical cost knowledge indices, it is important to remember that an increase in one unit means changing from not deprived status to a deprived status. Thus, for zero maternal education, being deprived in child health precautions increases the probability of being severely malnourished by 42.6 percent for male children and 46.1 percent for female children. Similarly being deprived in medical cost knowledge reduces the probability of having normal health by 33.1 percent for male children and 13.6 percent for female children.

Male children seem to be more sensitive to child health precautions and medical cost knowledge while female children more to health seeking practices. For zero maternal education, being deprived in health seeking practices increases the probability of being severely malnourished by 63.4 percent while decreases the probability of normal growth by 36.2 percent for female children (comparable estimate are 5.6 percent and 5.0 percent for male children, although insignificant). In other words policies looking to improve the nutritional status of female children *vis-a-vis* male children should focus on health-seeking practices.

The disparity in the results for female children compared to male children is quite noticeable. It is possible that prevailing socio-economic norms in Bangladesh, which tend to place a greater value on male offspring, leads to boys receiving more attention and a better quality of care as infants, which positively affects their health, physical robustness and immunity later on. It is an issue which requires further inquiry.

For mothers with primary education however, being deprived in health seeking practices increases the probability of being severely malnourished by 5.5 percent (for zero maternal education this value was 63.4 percent) while decreases the probability of normal growth by 11.3 percent (for zero maternal education this value was 36.2 percent) for female children. This sharp decline in the probability estimates indicate that education does offset the negative impact caused due to being deprived in health seeking practices for female children.

## Discussion

Our indices reflect that maternal health knowledge can have differential impact on child health based on gender; however, higher maternal education does reduce the gender bias. Impact of taking more child health precautions (vitamin supplements, immunizations, iodized salt and adequate adult support) seem to be imparted more on male children. Interestingly, children with older siblings are found to have better nutritional status, which we speculate to be a reflection of better care and/or increased family earnings.

## Conclusion

Our study considered the role of selected child, household and maternal factors on child nutritional status in an urban slum settlement in Bangladesh. Unlike many studies, our dataset gave us the advantage of constructing health knowledge indices of health-seeking practices, child health precautions and medical cost knowledge thereby understanding their role towards child health. We use available anthropometric weight-for-age nutrition status of the children for an ordered logistic analysis. It should be noted that while findings based on different anthropometric measures have not been robust in the literature, we could not compare to other anthropometric measures due to data unavailability.

Based on arguments that the gender dummy does not fully capture the gender differentials, we carry out gender specific regressions in comparison to a total regression. Our results reassert that male and female children do indeed have separate nutritional functions. Finally we use two instruments for income: per capita income and household assets, the latter being a crude proxy for permanent income. The main findings from the study are summarized below:

- The child health precautions, health seeking practices and medical cost knowledge indices, each reflecting a separate pathway for health knowledge, capture a portion of the total education effect, enough to make it insignificant for our study sample. We also found health knowledge to have differential impacts on child health. While male children are more sensitive to child health precautions and medical cost knowledge of mothers, female children (while also responsive to the two mentioned) are more sensitive to health-seeking practices. So policy makers looking to improve the nutritional status of female children *vis-a-vis* male children should promote health-seeking practices.
- Increase in per capita income improves the health status of all children. However, substituting per capita income with household assets does not lead to the same result. Assuming household assets to be a crude measure of permanent income, this casts doubt about the robustness of income explaining child health.
- The effect of maternal education on child nutritional status is gender specific. Our result suggests that better maternal literacy improves health of male children at the cost of the health of female children. This may be due to the local labor market where male members have better job prospects than female members.
- Finally, children with older siblings have better nutritional status, and this is significant for both male and female children. We reason that older siblings help either through increased care, participating in household chores or by earning which in turn translate to better health for their younger siblings.

## Footnotes

1 It is worth mentioning that the dataset contained only the nutritional status information of the children and not the continuous weight-for-age measure, hence the ordered variable was used.

2 Result not reported in table 03.

3 This is for households not deprived in child health precautions and health seeking practices, as well as zero per capita income. Comparable estimates for one unit increase in education with mean per capita income, which is 1.375 (BDT 1,375) results in a decrease in the probability of being severely malnourished by 43.5 percent for male children while an increase in the probability by 60.1 percent for female children. Note however, that the corresponding interaction terms are not statistically significant.
